# Malnutrition and Mold: When the Body Starves, the Fungus Thrives

**DOI:** 10.7759/cureus.92510

**Published:** 2025-09-17

**Authors:** Sidra Naeem, Hugo Farne, Meg Coleman

**Affiliations:** 1 Internal Medicine, Imperial College Healthcare NHS Trust, London, GBR; 2 Cardiology, The Hillingdon Hospitals NHS Foundation Trust, London, GBR; 3 Pulmonology, Imperial College Healthcare NHS Trust, London, GBR

**Keywords:** aspergillus fumigatus, cavitary lung disease, immunosuppression, malnutrition, opportunistic fungal infection

## Abstract

Chronic pulmonary aspergillosis (CPA) is typically associated with structural lung disease, such as prior tuberculosis, chronic obstructive pulmonary disease, or immunosuppression. Malnutrition, however, is an under-recognized contributor to immune dysfunction and may predispose to fungal infections. We present two cases of CPA in severely malnourished individuals without significant pre-existing pulmonary pathology. The first case involved a 41-year-old woman with anorexia nervosa, frailty, and osteoporosis who was incidentally found to have right-sided cavitary lesions on trauma imaging following a road accident. Despite positive *Aspergillus *IgG serology, she remained asymptomatic, and a conservative approach with nutritional rehabilitation was adopted. The second case involved a 41-year-old man with long-standing dietary restriction, who developed a chronic cough and was found to have a right apical cavitary lesion with positive *Aspergillus *culture. Antifungal therapy was initiated but discontinued due to side effects, and supportive care with physiotherapy and nutritional input was emphasized. These cases highlight malnutrition as a significant and potentially modifiable risk factor in CPA pathogenesis, warranting greater clinical awareness.

## Introduction

Chronic pulmonary aspergillosis (CPA) is a progressive fungal disease caused primarily by *Aspergillus fumigatus*, affecting an estimated three million individuals worldwide and associated with significant morbidity and mortality [1]. CPA can affect individuals who are immunocompetent or mildly immunocompromised and have underlying lung disease, such as chronic obstructive pulmonary disease, sequelae of tuberculosis, nontuberculous mycobacterial infections, or lung cancer [2].

Aspergillosis is caused predominantly by *Aspergillus fumigatus*, the most frequent mould infection of the lungs. The spectrum of the disease is broad and depends on the immune status of the host. There are distinct patterns of CPA with overlapping clinical presentations ranging from simple aspergilloma (a fungus ball within an existing cavity) to chronic cavitary, fibrosing, and microinvasive forms of pulmonary aspergillosis [3]. The diagnosis is based on a combination of radiological findings, microbiological or serological evidence of *Aspergillus *infection, and symptoms persisting for at least three months.

While structural lung disease and immunosuppression remain the strongest recognized risk factors, malnutrition is increasingly being recognized as an important but underappreciated contributor to immune dysfunction [4]. Malnutrition impairs both innate and adaptive immune responses, predisposing to prolonged or opportunistic infections, yet its role in fungal diseases such as CPA remains poorly defined [5].

We describe two unusual cases of CPA occurring in severely malnourished patients without significant pre-existing pulmonary disease. These cases highlight the potential importance of nutritional status in CPA pathogenesis and the need to consider host nutritional state when evaluating patients with unexplained cavitary lung disease.

## Case presentation

Case one

**Figure 1:**
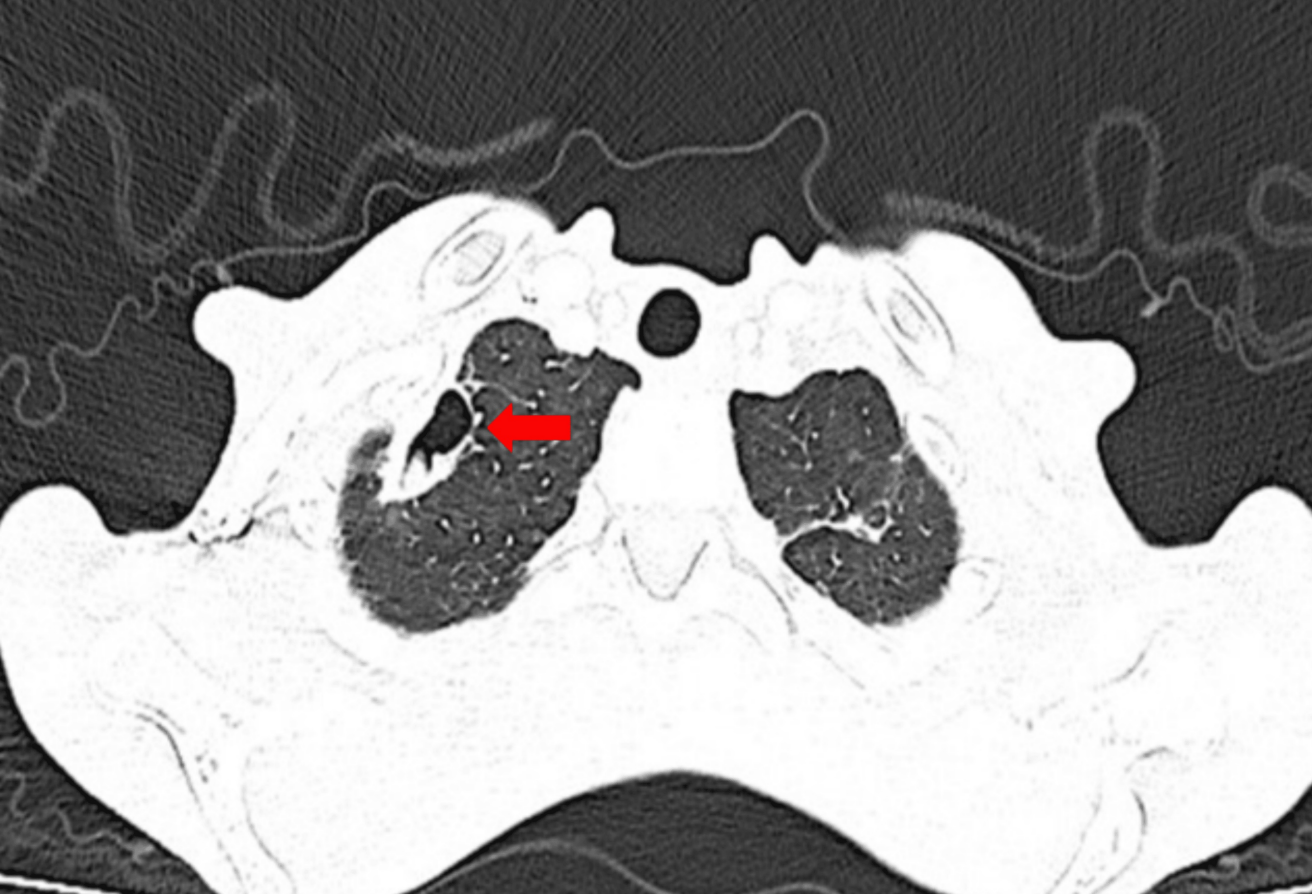
CT thorax showing two cavitating lesions with dependent fluid in the right lung Axial CT thorax (lung window) showing a 2.9 × 1.6 cm cavitary lesion in the right lung apex (arrow) with dependent fluid (case 1)

**Figure 2:**
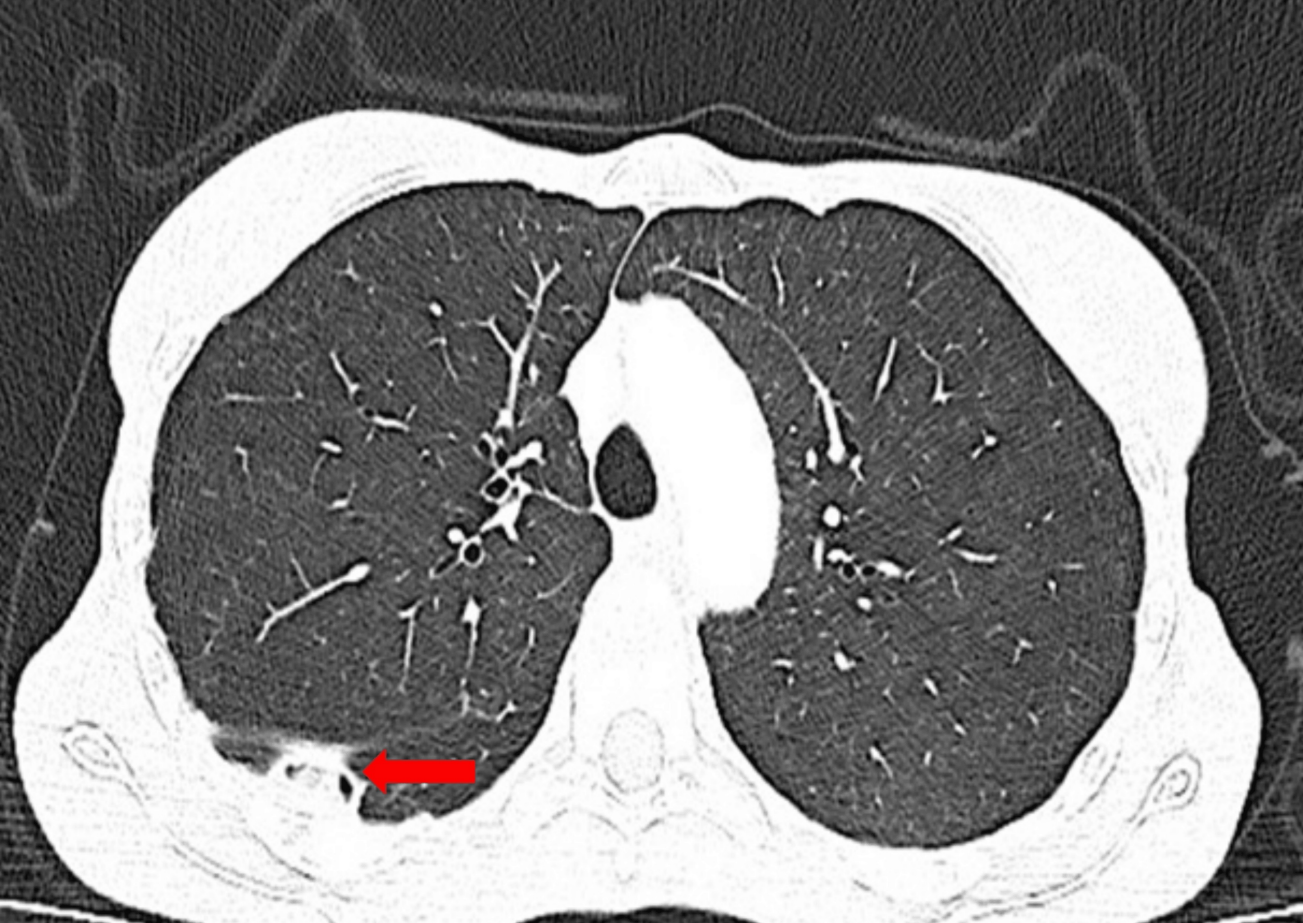
CT thorax showing two cavitating lesions with dependent fluid in the right lung Axial CT thorax (lung window) showing a 2.5 × 1.4 cm cavitary lesion in the superior segment of the right lower lobe (arrow) with dependent fluid (case 1)

A 41-year-old woman with a history of anorexia nervosa, osteoporosis, frailty, and body mass index (BMI) of 14.5kg/m2 had a computer tomography (CT) scan following a scooter-related road traffic accident that incidentally revealed two cavitating lesions in her right lung (Figures 1, 2). She was systemically well with no respiratory symptoms. 

She denied any history of tuberculosis, pneumonia, asthma, or atopy and was a lifelong nonsmoker, although she did report that respiratory viral infections resulted in prolonged cough. She also had historical neutropenia (thought to be nutritional), but recent counts were normal. 

She proceeded to have a bronchoalveolar lavage (BAL) to further investigate these lesions, with cultures negative for bacterial, fungal, and mycobacterial organisms, negative tuberculosis (TB) polymerase chain reaction (PCR) and respiratory viral PCR, and no malignant cells on cytology. She had a raised *Aspergillus fumigatus* IgG (71.7 mg/L; normal <40mg/L), with negative *Aspergillus fumigatus* IgE and normal total IgE (36.8 IU/mL; normal <100IU/mL).

She met diagnostic criteria for CPA [1]. However, given the lack of symptoms, negative BAL culture results, and only moderately raised IgG, the decision was made to monitor her rather than treat with an antifungal, alongside continued nutritional rehabilitation under her therapy team.

Case two

A 44-year-old man with a history of poorly controlled asthma, generalized anxiety and significant weight loss with BMI 15.2 kg/m2, related to self-imposed dietary restrictions, (specifically oily food, trying to eliminate fat and oil from his diet and mainly followed a vegan diet), presented with a chronic cough of over one year's duration and few episodes of small volume hemoptysis (quantity varied from less than a teaspoon to a tablespoon). He adhered to a strict vegan diet and further avoided oils and fats, believing these exacerbated his cough and, thereafter, hemoptysis. He was a lifelong nonsmoker with no history of tuberculosis, malignancy, or recurrent lung infections. 

**Figure 3:**
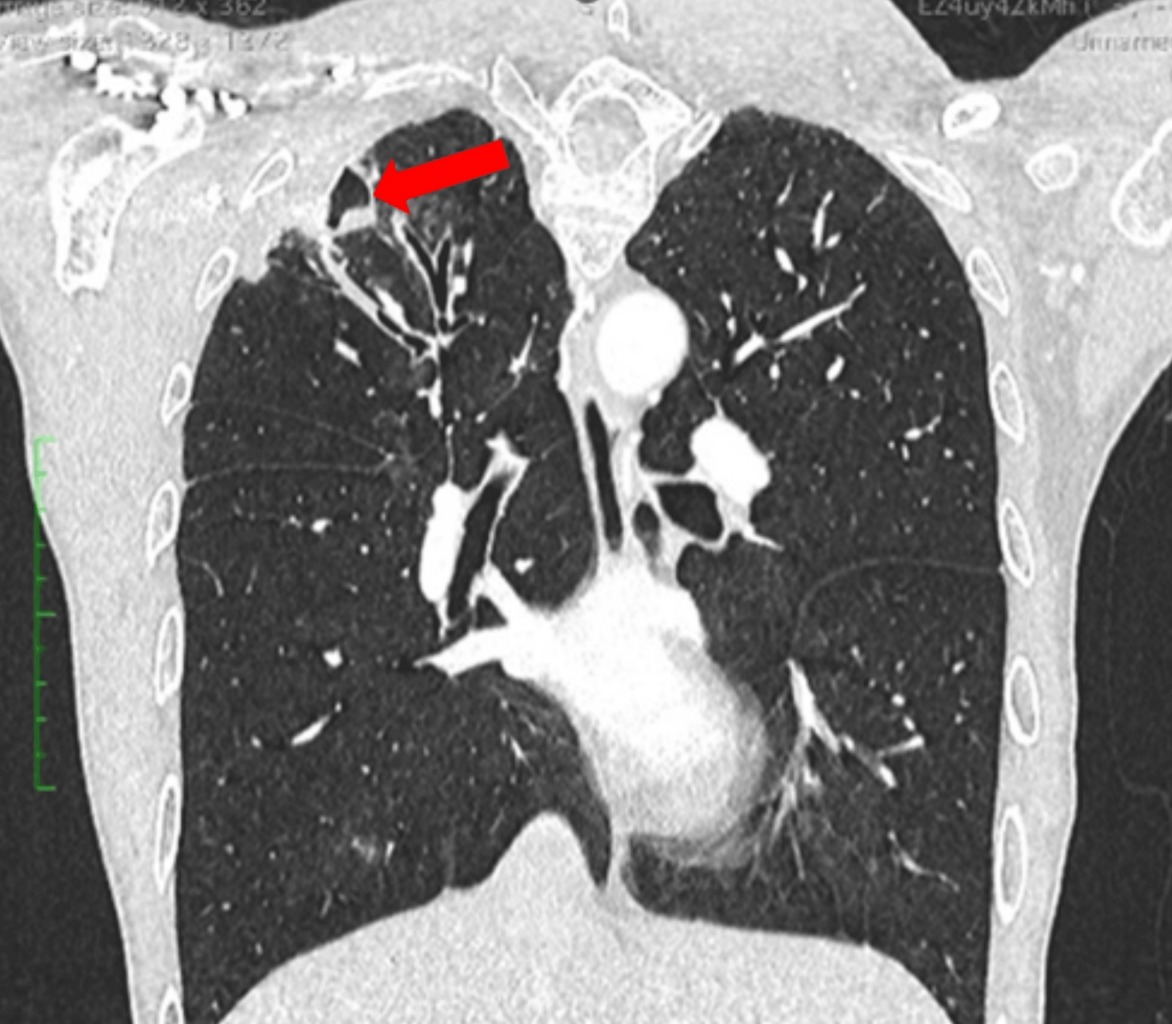
Chest CT revealed a thin-walled cavitary lesion with focal calcification in the posterolateral right apex, consistent with a chronic process Coronal CT thorax (lung window) demonstrating a right upper lobe cavitary lesion with thin wall and calcification (arrow) (case 2)

**Figure 4:**
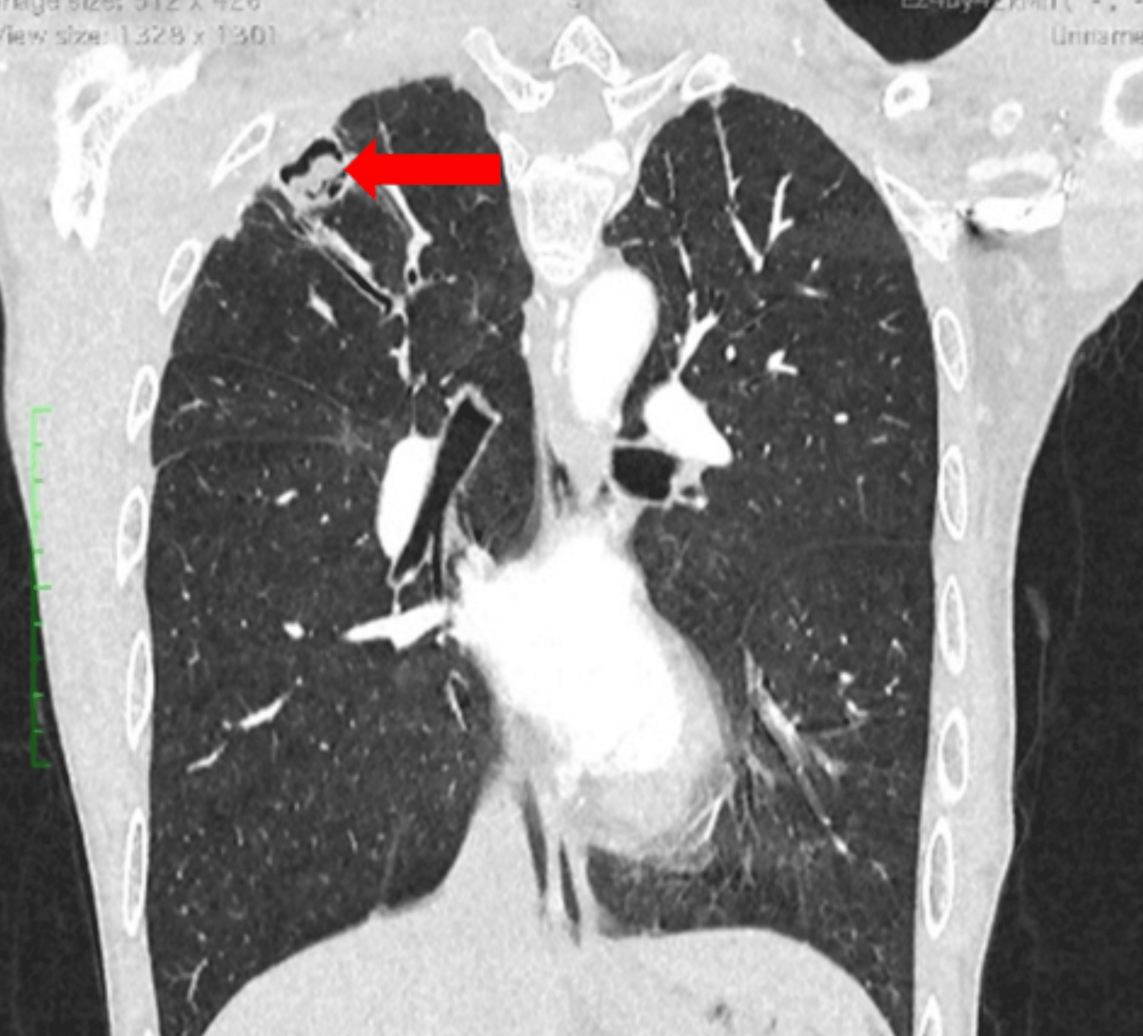
Image showing intracavitary body, suggestive of a mycetoma

Chest radiography and serial CT imaging revealed a right upper lobe cavitary lesion (Figures 3, 4), which was not present on imaging from 2012. The cavity had a thin wall with areas of calcification, implying chronicity. Despite the absence of classical risk factors such as structural lung disease or immunosuppression, his investigations were suspicious for respiratory fungal infection. He had a BAL with a fungal culture positive for *Aspergillus fumigatus*. Blood tests showed a raised *Aspergillus fumigatus* IgG (95.4mg/L), total IgE (869 IU/mL; previously >1000 IU/mL), and *Aspergillus fumigatus*-specific IgE (1.57 kU/L; normal <0.35kU/L). He therefore met diagnostic criteria for CPA. 

He was initiated on oral antifungal therapy (voriconazole, later switched to itraconazole due to intolerance) with improvement in his cough and hemoptysis. However, treatment was discontinued due to adverse effects (skin rash with voriconazole and hypokalemia with itraconazole) and compliance issues. The chest physiotherapy team highlighted the role of airway clearance in preventing sputum retention and secondary infection.

Despite longstanding undernutrition, the patient had not previously engaged with a dietitian or sought psychological support. He was advised to pursue multidisciplinary care, including nutritional rehabilitation and mental health support, to address modifiable contributors to disease progression and improve overall outcomes. He showed some weight gain of nearly 3 kgs on a follow-up appointment with treatment and improvement in diet; this is a continued work in progress.

## Discussion

CPA typically occurs in individuals with underlying lung disease, structural abnormalities, or immunodeficiency. However, both cases presented demonstrate CPA in non-classical hosts whose primary vulnerability was severe nutritional compromise, a risk factor that is underrecognized in clinical practice. When one looks for it, malnutrition is relatively common in CPA: 33-44% of patients with CPA also had malnutrition in a French national hospital database (2014-2018) [6]; 7-11% in an equivalent analysis of a Japanese national hospital database (2016-2022 )[7]. However, many will have had other comorbidities predisposing them to CPA, e.g., chronic respiratory disease; it is unclear how many were only comorbid with malnutrition.

The combination of characteristic clinical symptoms (e.g., productive cough, hemoptysis, fever, malaise, fatigue, weight loss), radiological findings (e.g., cavitating lesions), and either the presence of *Aspergillus fumigatus* IgG or the isolation of *Aspergillus fumigatus* from respiratory samples is highly indicative of CPA [1,2]. The two cases above fulfilled this criterion.

Malnutrition impairs both innate and adaptive immunity, disrupting mucosal barriers, diminishing phagocyte function, and impairing cytokine signaling, thereby increasing susceptibility to fungal infections such as *Aspergillus fumigatus* [5]. Even in the absence of overt immunosuppression or structural lung damage, prolonged undernutrition can mimic an immunocompromised state, predisposing to opportunistic infections.

In case one, CPA was discovered incidentally in an asymptomatic woman with longstanding anorexia nervosa, frailty, and a history of neutropenia. The presence of cavitating lung lesions with elevated* Aspergillus fumigatus* IgG despite negative BAL fungal cultures fulfilled diagnostic criteria for CPA [8]. A conservative approach was justified in view of her asymptomatic status, focusing instead on nutritional rehabilitation.

In case two, the patient's restrictive vegan diet and avoidance of fats led to significant weight loss and micronutrient depletion. The subsequent development of CPA in the absence of prior pulmonary pathology highlights how dietary beliefs and behavioral health issues can intersect with infectious disease risk. Though antifungal therapy was initiated, drug intolerance and poor adherence limited treatment success [9]. Importantly, this patient had never engaged with nutritional or psychological services, underscoring missed opportunities for early, holistic intervention.

Emerging data link low BMI and hypoalbuminemia with worse outcomes in CPA and invasive aspergillosis [10]. Nutritional status is also known to correlate with immunoglobulin production, airway epithelial integrity, and treatment response [11]. These cases illustrate the need to broaden our conception of CPA risk beyond conventional immunosuppression to include malnutrition as a functional immunodeficiency.

While pulmonary aspergillosis is uncommon in immunocompetent hosts, clinicians should maintain a high index of suspicion in severely malnourished individuals presenting with respiratory symptoms and cavitary lung lesions.

Furthermore, they highlight the importance of multidisciplinary care, including respiratory, infectious disease, dietetic, and psychological input, for patients with CPA and nutritional vulnerability. Early recognition and nutritional support may not only improve overall resilience but also enhance antifungal therapy tolerability and reduce relapse risk [12].

## Conclusions

These two cases illustrate an atypical presentation of chronic pulmonary aspergillosis in patients without pre-existing pulmonary disease but with severe malnutrition. In the first case, cavitary lesions were discovered incidentally in an asymptomatic woman with anorexia nervosa, while in the second, a man with longstanding dietary restriction presented with chronic cough and a culture-proven *Aspergillus infection*. Both cases underscore how malnutrition, even in the absence of classical respiratory risk factors, may create a vulnerable host environment for fungal disease.

The key lesson from these cases is the importance of considering nutritional status as part of the host risk profile when evaluating unexplained cavitary lung lesions. While antifungal therapy remains the mainstay of treatment in symptomatic disease, addressing underlying nutritional deficits through multidisciplinary input is equally critical in modifying disease progression and improving outcomes. These reports highlight the need for greater awareness of malnutrition as a modifiable risk factor in the pathogenesis of CPA.
